# Seasonal dynamics and niches of three vector chigger species at a focus of scrub typhus in southwest China

**DOI:** 10.3389/fvets.2025.1637410

**Published:** 2025-09-29

**Authors:** Yan Lv, Peng-Wu Yin, Xian-Guo Guo, Rong Fan, Cheng-Fu Zhao, Zhi-Wei Zhang, Ya-Fei Zhao, Lei Zhang

**Affiliations:** Yunnan Provincial Key Laboratory for Zoonosis Control and Prevention, Institute of Pathogens and Vectors, Dali University, Dali, Yunnan, China

**Keywords:** chigger mite, vector of scrub typhus, seasonal fluctuation, host selection, *Leptotrombidium deliense*, *Leptotrombidium scutellare*, *Leptotrombidium imphalum*

## Abstract

**Objective:**

The present study aims to investigate the seasonal dynamics of main vectors of scrub typhus, and thereby provide scientific information for the surveillance and control of the disease.

**Methods:**

A field investigation lasting for 12 consecutive months was conducted at a fixed survey site (Waxi Village) in Binchuan County (a focus of scrub typhus), Yunnan Province of southwest China between 2019 and 2020. Based on the taxonomic identification of all collected chigger mites, the three vector chigger species of scrub typhus (*Leptotrombidium deliense, L. scutellare* and *L. imphalum*) were screened out as the object of this study. The constituent ratio (*C_r_*), prevalence (*P_M_*), mean abundance (*MA*), and mean intensity (MI) were calculated to reflect the mite infestation status. The Levins’ niche breadth (*Bi*) and Pianka’s proportional similarity ratio (*O_ij_*) were used to calculate the ecological niche breadth and niche overlap of chiggers on different host species and temporal series.

**Results:**

These three vector chigger species were the dominant mites at the survey site, accounting for 60.72% of the total 115 chigger species (*C_r_*=60.72%, 132,160/217,671). In seasonal fluctuations, *L. deliense* showed two peaks in summer and autumn, and the highest peak was in August of summer with highest infestation indexes (*C_r_*=44.29%, *MA*=322.48, *MI*=422.56) on the hosts. *Leptotrombidium imphalum* mainly appeared in summer and autumn, and peaked in September (*C_r_*, *MA* and *MI*) and October (*P_M_*) of early autumn. *Leptotrombidium scutellare* mainly appeared in November, December and January, and peaked in November of late autumn (*C_r_*=66.09%) and December of early winter (*C_r_*=33.83%). *Leptotrombidium deliense* had the widest temporal niche (*Bi*=0.248) and *L. scutellare* had the widest host niche (*Bi*=0.268). *Leptotrombidium deliense* and *L. imphalum* had the highest temporal niche overlap (*O_ij_*=0.715) and host niche overlap (*O_ij_*=0.986). The monthly average temperature significantly affected the seasonal fluctuation of *L. scutellare*.

**Conclusion:**

The three vector chigger species can parasitize a wide range of hosts with low host specificity, and their coexistence as the dominant mites at the survey site increases the potential risk of transmission and focus persistence of relevant mite-borne zoonoses. *Leptotrombidium deliense* and *L. imphalum* have similar seasonal distribution patterns (summer-autumn type) and host selection, and *L. scutellare* has a different type of seasonal fluctuation, autumn-winter type. The temporal and host niches of *L. scutellare* are very different from those of *L. deliense* and *L. imphalum*. The temperature is the most important climate factor that drives the seasonal dynamics of vector chiggers.

## 1 Introduction

Chiggers (chigger mites) usually refer to the larval stage of trombiculid mites. The life cycle of trombiculid mites is complex with several stages and only the larval stage (chigger) is the ectoparasite of other animals. Rodents and other small mammals are the most common hosts of chiggers (1, 2). To date, there have been more than 3,000 chigger species recorded globally, and over 500 species documented in China (3, 4). Chiggers are the exclusive vector of *Orientia tsutsugamushi* (Ot), the causative agent of scrub typhus (tsutsugamushi disease). Besides, some chiggers (e. g. *L. scutellare*) can serve as the potential vector of *Hantavirus* (HV), the pathogen of hemorrhagic fever with renal syndrome (HFRS) (5, 6). Both scrub typhus and HFRS are zoonotic diseases (zoonoses), which can be transmitted among wild animals (especially rodents) and even from wild animals to human beings through the biting activity of chigger mites (7–9). Nowadays, scrub typhus has become a serious public health problem with more than one million cases reported globally each year (10). In China, the epidemic foci of scrub typhus have expanded from 26 counties (or districts) in 16 provincial regions in 2006 to 1,150 counties (or districts) in 29 provincial regions in 2023, with the number of reported cases rising by 25.80 times over the 18-year period (11). Yunnan Province of southwest China is a main focus of scrub typhus. Of 283,273 cases of scrub typhus reported in China between 2006 and 2023, 29.93% (84,795 cases) were from Yunnan, which ranked first among all provincial regions in the country (11). Binchuan County of Dali Prefecture, the survey site of the present study, is an important focus of scrub typhus in Yunnan, and an outbreak of scrub typhus was once reported in the county (12).

Recent studies have shown that the species composition of chigger mites is complex in Yunnan, with coexistence of multiple vector species such as *L. deliense*, *L. scutellare*, *L. sialkotense* and *L. imphalum* (13–15). There are often seasonal fluctuations in the population of chigger mites, which in turn affects seasonal changes in the incidence of scrub typhus (16–18). Based on a consecutive 12-month investigation and taxonomic identification of chiggers at a fixed survey site, Waxi Village of Bingchuan County, Dali Prefecture of Yunnan Province where both scrub typhus and HFRS are prevalent with high incidence of the diseases (11, 19, 20), this paper reported the seasonal fluctuations and ecological niches (temporal niche and host niche) of three vector chigger species, *L. deliense*, *L. scutellare* and *L. imphalum*. Of the three vector chigger species, *L. deliense* and *L. scutellare* are the two most important vectors of scrub typhus in China (5, 8, 17), and *L. scutellare* is also a potential vector of HFRS (5, 6). *Leptotrombidium imphalum* has been confirmed as one of the vectors of scrub typhus in Thailand (21, 22), and it is an important potential vector of the disease in China (23, 24). The present study is an attempt to enrich the knowledge about the related vector chigger species and provide the scientific information for the surveillance and control of related mite-borne diseases.

## 2 Materials and methods

### 2.1 Investigation, collection and identification of chigger mites

**Figure 1:**
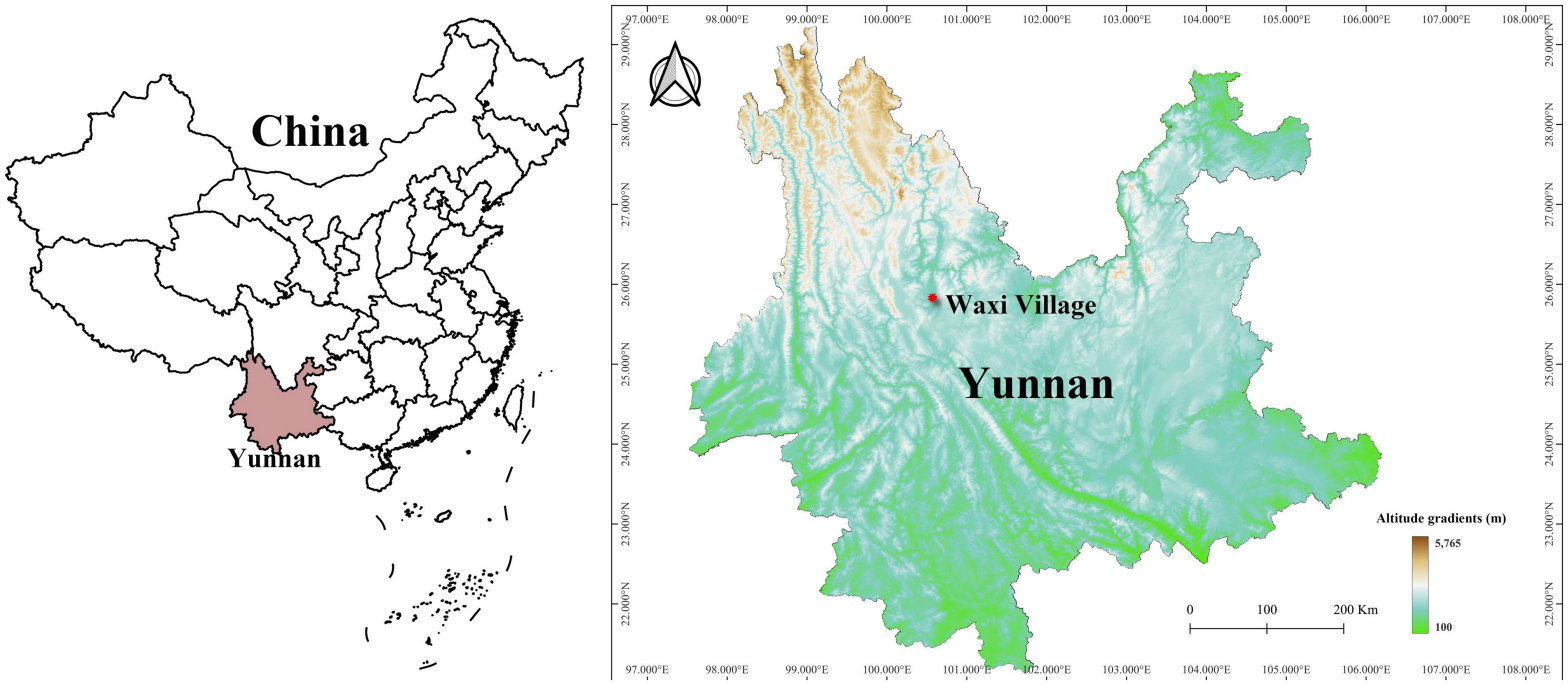
Map of China and Yunnan Province showing the location of the fixed survey site (Waxi Village) in Binchuan County, Dali Prefecture, Yunnan Province of southwest China (2020–2021).

A consecutive 12-month investigation was carried out at Waxi Village, Binchuan County, Dali Prefecture, Yunnan Province of southwest China (25^°^43′ N, 100^°^24′ E) from November 2020 to October 2021 (Figure 1). In China, the four seasons of a year are divided as follows: March to May is spring, June to August is summer, September to November is autumn, and December to February is winter. The fixed survey site (Waxi village) is located in southern subtropical areas, with a typical subtropical monsoon climate (25). Each month at the fixed survey site, 150–200 mouse traps (18 × 12 × 9 cm, Guixi Mousetrap Apparatus Factory, Guixi, Jiangxi, China) were placed to capture rodents and other sympatric small mammals (hosts) in the late afternoon or evening. Considering the varying host densities in different months, the field investigation for each month lasted for 7–10 days and the number of mouse traps were adjusted accordingly to ensure a sufficient number of host samples (at least 100 hosts per month). The captured hosts were collected with white cloth bags in the following morning, and then were transported to the temporary field laboratory for the collection of chiggers. Each animal host was routinely anesthetized with ether and placed in a large white square plate to collect chiggers on the body surface of each host, especially the auricle, the opening of external auditory canal, groin, perianal area, and other thin tender skin areas where chiggers are often attached. The collected chiggers were preserved in 70% or 75% ethanol for fixation (13, 26).

Chiggers have to be identified under a microscope because of their minute structure and abundant species, which is a big challenge. In the laboratory, the collected chiggers were mounted onto glass slides with Hoyer’s medium (Hoyer’s solution). After the process of dehydration, transparency and drying, each mounted chigger specimen was carefully observed and relevant morphological structures were measured under the higher power lens and oil lens of a microscope (Olympus Corporation, Tokyo, Japan). Under the microscope, all chigger specimens were identified to species one by one with the help of relevant taxonomic books, literature and taxonomic keys (1, 27, 28). After all chigger specimens were accurately identified, three vector chigger species (*L. deliense*, *L. scutellare* and *L. imphalum*) were screened out as the object of the present study. The capture and use of animals were approved by the local wildlife affairs authority and the Ethics Committee of Dali University.

### 2.2 Chigger infestation statistics

The constituent ratio (*C_r_*) was used for calculating the percentage of chiggers and its associated host species. The infestation prevalence (*P_M_*) was calculated for the percentage of infested hosts with chiggers, and the mean abundance (*MA*) and mean intensity (*MI*) were used for the average number of chiggers per examined host and the average number of chiggers per infested host, respectively (26). Pearson correlation coefficient (*r*) was used to analyze the relationships between the monthly infestation indexes (*C_r_*, *P_M_*, *MA* and *MI*) of vector chigger species and climatic factors (temperature, rainfall and humidity), and between the monthly infestation indexes and monthly human cases of scrub typhus.

### 2.3 Ecological niche analysis

Levins’ niche breadth (*B_i_*) and Pianka’s proportional similarity ratio (*O_ij_*) were used to calculate the ecological niche breadth and niche overlap of chiggers on different host species and temporal series (13, 29, 30).

$$B_{i}=\frac{1}{S\sum_{h=1}^{S}{P_{\mathrm{ih}}}^{2}}$$

$$\mathrm{Oij}=\frac{\sum_{h=1}^{S}P_{\mathrm{ih}}P_{\mathrm{jh}}}{\sqrt{\sum_{h=1}^{S}{P_{\mathrm{ih}}}^{2}\sum_{h=1}^{S}{P_{\mathrm{jh}}}^{2}}}$$

In the above formulae, *B_i_* represents the niche breadth of a certain chigger species (species *i*), and *O_ij_* stands for the niche overlap index between any two chigger species, species *i* and *j*. The *P_ih_* and *P_jh_* stand for the proportion (constituent ratio, *C_r_*) of chigger species *i* or *j* on different host species (host series) or temporal series (time series), and *S* is the number of resource series (host series and time series). The values of *O_ij_* range from 0 to 1 [0, 1]. When the two chigger species have no resource (host resource or time resource) to share, their *O_ij_* is equal to 0 (a minimum value), and when they share all the available resources, their *O_ij_* reaches a maximum value of 1.

### 2.4 Source of relevant data

In the present study, the survey site (Waxi Village of Binchuan County) is located within the territory of Dali Prefecture in Yunnan Province of southwest China. In the analysis of correlation between the monthly chigger infestation and climatic factors, the meteorological data for the survey site were obtained from the website^1^ and the National Qinghai Tibet Plateau/Three Pole Environmental Data Center (https://data.tpdc.ac.cn) (31). As there were no monthly cases of scrub typhus available for the survey site (Waxi Village) and its affiliated county (Binchuan County), the monthly cases of the disease in Dali Prefecture and Yunnan Province from 2006 to 2022 were instead used to analyze the correlation between the monthly chigger infestation indexes and cases of scrub typhus (19). These monthly cases of scrub typhus were sourced from the published data by Li et al. (19) in 2024.

## 3 Results

### 3.1 Identification of chiggers and their small mammal hosts

A consecutive 12-month investigation was conducted at the fixed survey site (Waxi Village) between 2020 and 2021 (Figure 1). A total of 1,329 small mammal hosts captured were identified as three orders, five families, 12 genera and 18 species. Among the 18 host species (1,329 individuals), there were 11 species (1,191 individuals) of rodents belonging to one family (Muridae) and five genera in the order Rodentia (Table 1). Rodents (Rodentia) accounted for 61.11% of the total host species (*C_r_* = 61.11%, 11/18) and 89.62% of the total host individuals (*C_r_* = 89.62%, 1,191/1,329). A total of 217,671 chiggers collected from the body surface of 1,329 hosts were identified as one family (Trombiculidae), 13 genera and 115 species. The 217,671 identified chiggers did not include 5,454 unidentified chigger specimens. These 5,454 unidentified chigger specimens were not identified to species level because of structural damage, dirt coverage, ambiguous structure, or suspicion of new species.

**Table 1:** Taxonomic identification of rodents and other sympatric small animals (hosts of chiggers) at Waxi Village of Binchuan County, Dali Prefecture, Yunnan Province of southwest China (2020–2021).

Orders	Families	Numbers and constituent ratios of Genera		Numbers and constituent ratios of species		Numbers and constituent ratios of individuals	
		No.	*C_r_*, %	No.	*C_r_*, %	No.	*C_r_*, %
Rodentia	Muridae	5	41.67	11	61.11	1,191	89.62
	Cricetidae	1	8.33	1	5.56	27	2.03
	Sciuridae	3	25.00	3	16.67	45	3.39
Eulipotyphla	Soricidae	2	16.67	2	11.11	4	0.30
Scandentia	Tupaiidae	1	8.33	1	5.56	62	4.67
Total	5 families	12	100.00	18	100.00	1,329	100.00

### 3.2 Distribution of three vector chigger species on different hosts

**Figure 2:**
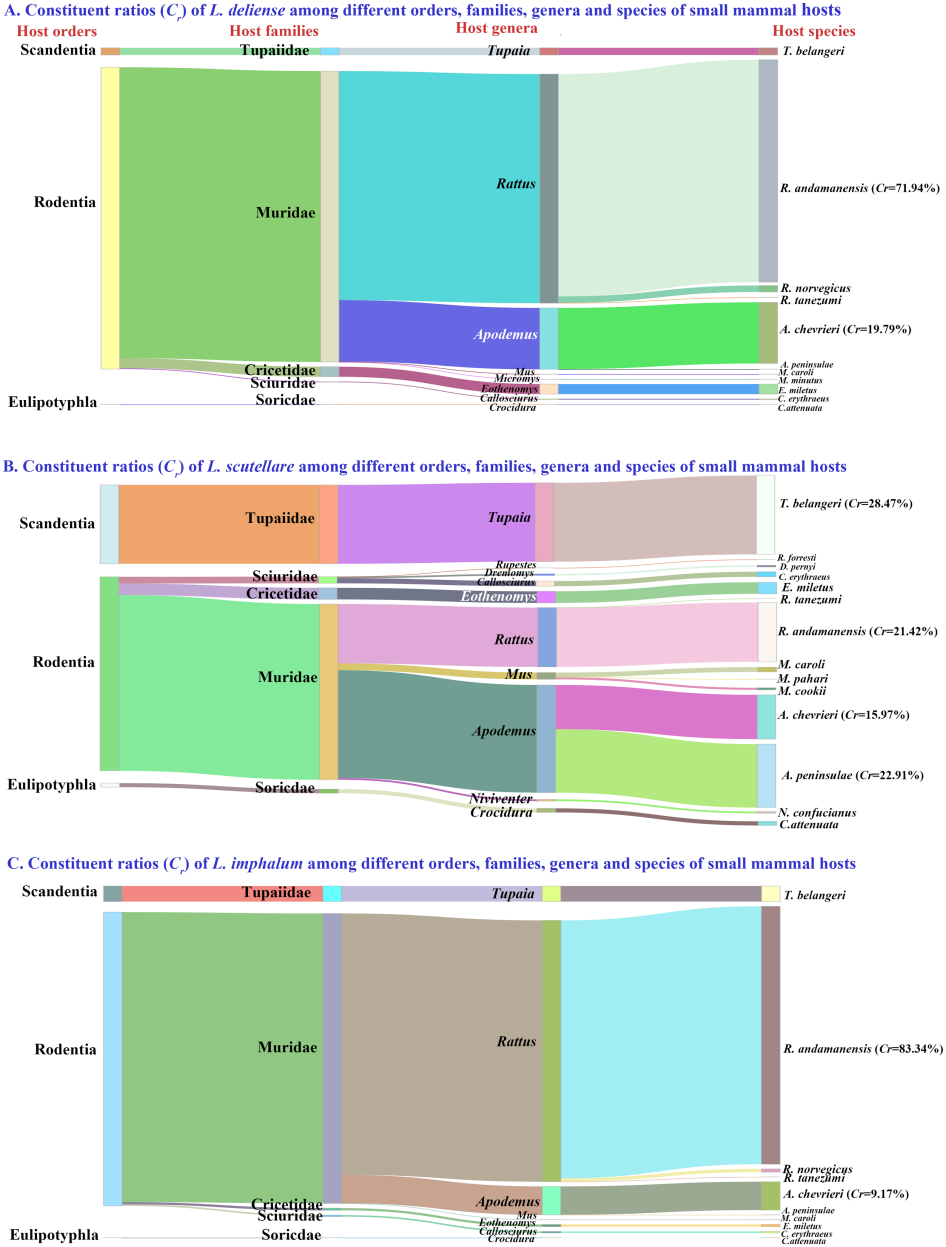
Visualization of constituent ratios (*C_r_*) of three vector chigger species (*L. delicense*, *L. scutellare* and *L. imphalum*) among different orders, families, genera and species of small mammal hosts at Waxi Village of Binchuan County, Dali Prefecture, Yunnan Province of southwest China (2020–2021). The shade width indicates the constituent ratio of the corresponding chigger species on a certain order, family, genus or species of small mammal hosts. The adjacent shades originate from a certain host taxon, an order, family or genus.

Of the 217,671 chiggers (115 species) identified, 83,008 *L. deliense*, 24,839 *L. scutellare* and 24,313 *L. imphalum* were screened out, and the number of these three vector chigger species (132,160) accounted for 60.72% of the total chigger mites (*C_r_* = 60.72%, 132,160/217,671). The hosts of the three vector chigger species crossed different orders, families, genera and species of small mammals, and rodents (the order Rodentia) were the main hosts of the chiggers with the majority of the chiggers identified from different taxonomic levels of rodents (Figure 2). For example, 83,008 *L. deliense* mites were identified from 11 species (1,016 individuals) of hosts, 71.94% of *L. deliense* (*C_r_* = 71.94%, 59,716/83,008) were found on the rat *Rattus andamanensis* (Figure 2A). The 24,839 *L. scutellare* mites came from 14 species (1,132 individuals) of hosts, and three species of small mammals were the main hosts of *L. scutellare* with 28.47% of *L. scutellare* mites (*C_r_* = 28.47%, 7,071/24,839) found on the tree shrew (*Tupaia belangeri*), 22.91% of the mites (*C_r_* = 22.91%, 5,690/24,839) on the mouse *Apodemus peninsulae*, and 21.42% of the mites (*C_r_* = 21.42%, 5,320/24,839) on the rat *R. andamanensis* (Figure 2B). The hosts of 24,313 *L. imphalum* involved 10 species (1,154 individuals) of small mammals, and 83.34% of *L. imphalum* (*C_r_* = 83.34%, 20,263/24,313) were found on *R. andamanensis* (Figure 2C).

### 3.3 Seasonal fluctuations of three vector chigger species

**Figure 3:**
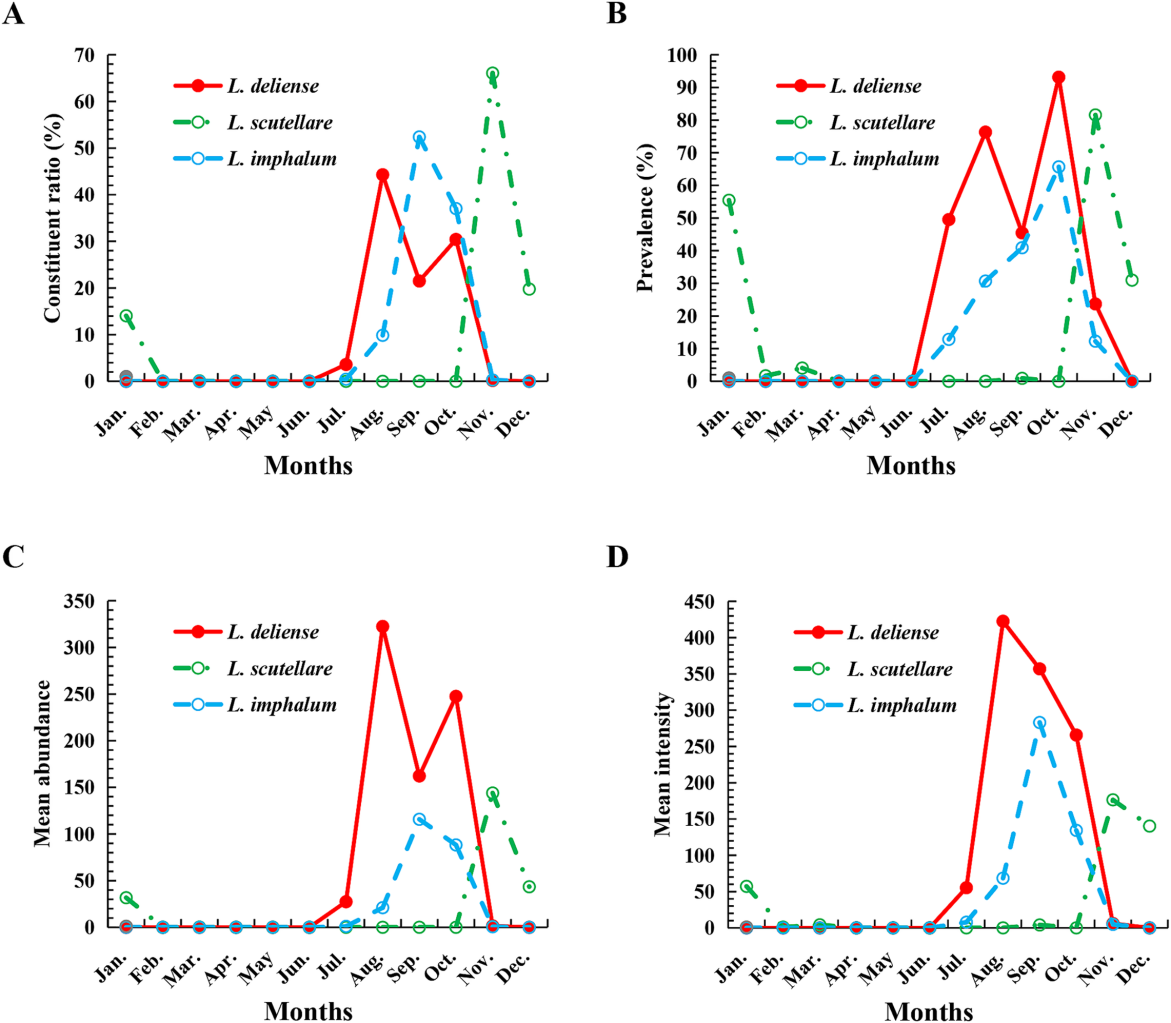
Monthly fluctuations of constituent ratios (*C_r_*), prevalence (*P_M_*), mean abundance (*MA*) and mean intensity (*MI*) of three vector chigger species (*L. deliense*, *L. scutellare* and *L. imphalum*) at Waxi Village of Binchuan County, Dali Prefecture, Yunnan Province of southwest China (2020–2021). (**A**) Monthly fluctuation of constituent ratios (*C_r_*) of chiggers; (**B**) Monthly fluctuation of prevalence (*P_M_*) of chiggers; (**C**) Monthly fluctuation of mean abundance (*MA*) of chiggers; (**D**) Monthly fluctuation of mean intensity (*MI*) of chiggers.

**Figure 4:**
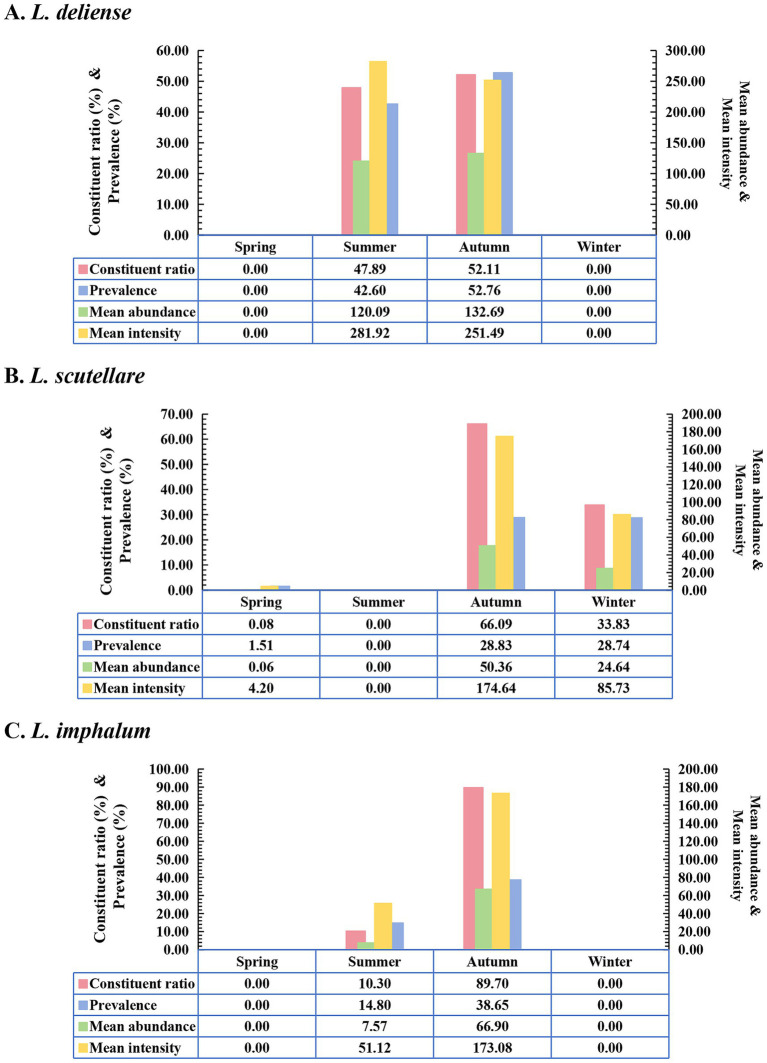
Seasonal fluctuations of constituent ratios (*C_r_*), prevalence (*P_M_*), mean abundance (*MA*) and mean intensity (*MI*) of three vector chigger species (*L. deliense*, *L. scutellare* and *L. imphalum*) at Waxi Village of Binchuan County, Dali Prefecture, Yunnan Province of southwest China (2020–2021). (**A**) Seasonal fluctuation of *L. deliense*; (**B**) Seasonal fluctuation of *L. scutellare*; (**C**) Seasonal fluctuation of *L. imphalum*.

Based on the monthly investigations conducted at the fixed survey site (Waxi Village) from November 2020 to October 2021, the seasonal fluctuations of three vector chigger species (*L. deliense*, *L. scutellare* and *L. imphalum*) were analyzed. Different chigger species had different patterns of seasonal fluctuations. *Leptotrombidium deliense* showed two peaks for its monthly fluctuation, and the highest peak (the first peak) was in August of summer with highest constituent ratio (*C_r_* = 44.29%), infestation mean abundance (*MA* = 322.48 mites/examined host) and mean intensity (*MI* = 422.56 mites/infested host) on its small mammal hosts. The second peak of *L. deliense* occurred in October of autumn (*C_r_* = 30.42%, *MA* = 247.54 and *MI* = 265.78) with a sharp decline in November, and no *L. deliense* was found in December of winter (Table 2, Figure 3). In four seasons of a year, *L. deliense* also showed two peaks for its seasonal fluctuation with the first peak in summer (June, July and August, *C_r_* = 47.89%) and the second peak in autumn (September, October and November, *C_r_* = 52.11%) (Table 2, Figure 4A).

**Table 2:** Monthly fluctuations of three vector chigger species (*L. deliense*, *L. scutellare* and *L. imphalum*) at Waxi Village of Binchuan County, Dali Prefecture, Yunnan Province of southwest China (2020–2021).

Examined hosts	Jan.	Feb.	Mar.	Apr.	May	Jun.	Jul.	Aug.	Sep.	Oct.	Nov.	Dec.
	110	118	123	104	104	108	109	114	110	102	114	113
*L. deliense*												
Individuals	0	0	0	0	0	0	2,988	36,763	17,848	25,249	160	0
*C_r_* (%)	0.00	0.00	0.00	0.00	0.00	0.00	3.60	44.29	21.50	30.42	0.19	0.00
*P_M_*	0.00	0.00	0.00	0.00	0.00	0.00	49.54	76.32	45.45	93.14	23.68	0.00
*MA*	0.00	0.00	0.00	0.00	0.00	0.00	27.41	322.48	162.25	247.54	1.40	0.00
*MI*							55.33	422.56	356.96	265.78	5.93	
*L. scutellare*												
Individuals	3,490	2	21	0	0	0	0	0	4	0	16,412	4,910
*C_r_* (%)	14.05	0.01	0.08	0.00	0.00	0.00	0.00	0.00	0.02	0.00	66.07	19.77
*P_M_*	55.45	1.69	4.07	0.00	0.00	0.00	0.00	0.00	0.91	0.00	81.58	30.97
*MA*	31.73	0.02	0.17	0.00	0.00	0.00	0.00	0.00	0.04	0.00	143.96	43.45
*MI*	57.21	1.00	4.20						4.00		176.47	140.29
*L. imphalum*												
Individuals	0	0	0	0	0	0	109	2,396	12,737	9,004	67	0
*C_r_* (%)	0.00	0.00	0.00	0.00	0.00	0.00	0.45	9.85	52.39	37.03	0.28	0.00
*P_M_*	0.00	0.00	0.00	0.00	0.00	0.00	12.84	30.70	40.91	65.69	12.28	0.00
*MA*	0.00	0.00	0.00	0.00	0.00	0.00	1.00	21.02	115.79	88.27	0.59	0.00
*MI*							7.79	68.46	283.04	134.39	4.79	

*Leptotrombidium scutellare* mainly appeared in November, December and January, with the highest constituent ratio (*C_r_* = 66.07%) and infestation indexes (*P_M_* = 81.58%, *MA* = 143.96 and *MI* = 176.47) in November for its monthly fluctuation. Except for November, December and January, there were very few or no *L. scutellare* in the remaining 9 months (Table 2, Figure 3). In four seasons of a year, *L. scutellare* peaked in late autumn (November, *C_r_* = 66.09%) and early winter (December, *C_r_* = 33.83%) for its seasonal fluctuation (Table 2, Figure 4B).

*Leptotrombidium imphalum* peaked in September (*C_r_* = 52.39%, *MA* = 115.79 and *MI* = 283.04) and October (*P_M_* = 65.69%) for its monthly fluctuation (Table 2, Figure 3). In four seasons of a year, *L. imphalum* mainly appeared in summer (*C_r_* = 10.30%) and autumn (*C_r_* = 89.70%), and peaked in early autumn (Table 2, Figure 4C).

### 3.4 Relationship between chigger seasonal fluctuations and climate factors

**Figure 5:**
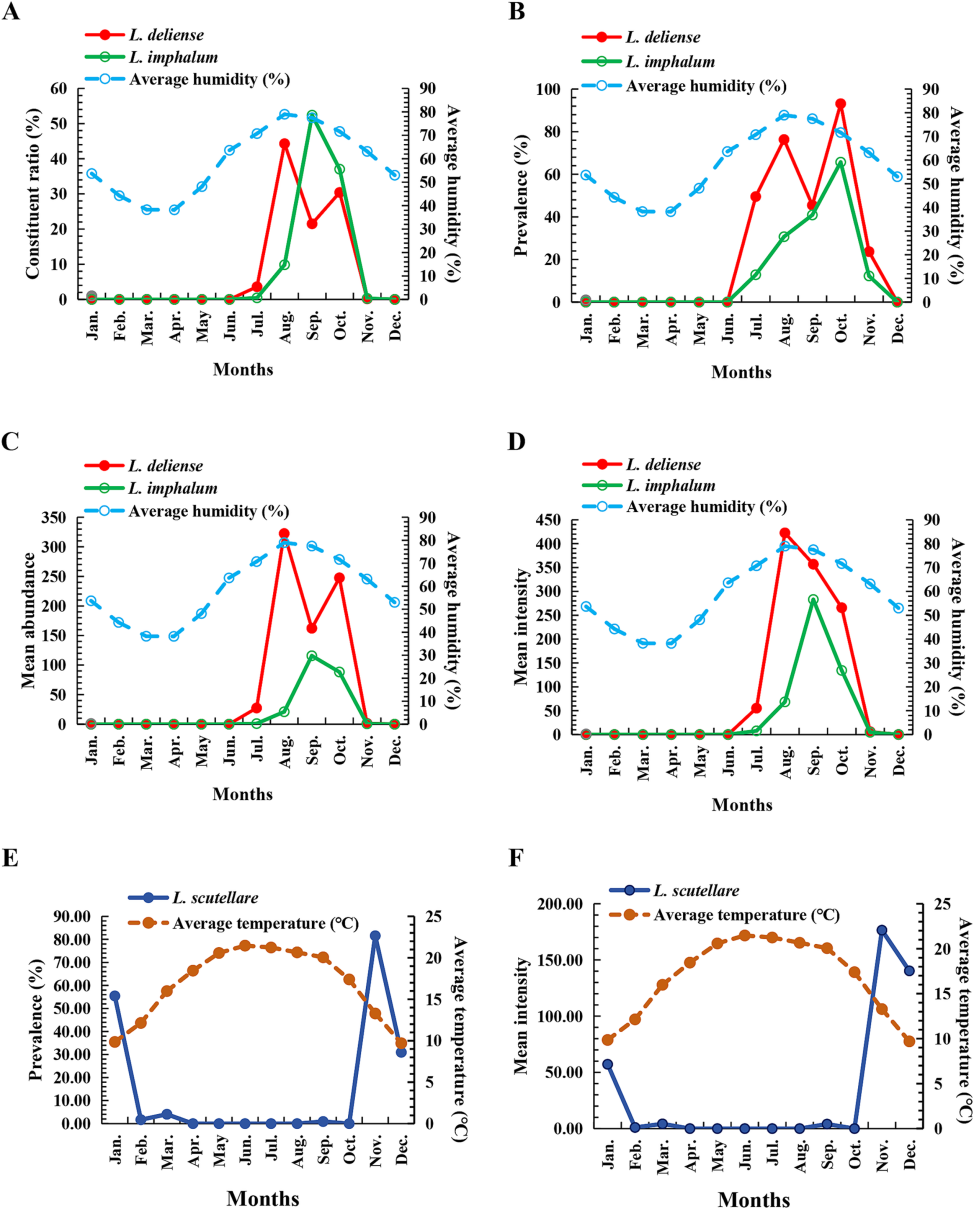
Monthly fluctuations of infestation indexes of three vector chigger species and climate factors at Waxi Village of Binchuan County, Dali Prefecture, Yunnan Province of southwest China (2020–2021). (**A–D**) Monthly fluctuations of infestation indexes (*C_r_*, *P_M_*, *MA* and *MI*) of two vector chigger species (*L. deliense* and *L. imphalum*) and monthly average humidity; (**E,F**) Monthly fluctuations of infestation indexes (*P_M_* and *MI*) of another vector chigger species (*L. scutellare*) and monthly average temperature.

Pearson correlation analysis was used to analyze the relationship between the infestation indexes (*C_r_*, *P_M_*, *MA* and *MI*) of three vector chigger species (*L. deliense*, *L. scutellare* and *L. imphalum*) on their small mammal hosts and three climate factors, the monthly average temperature, monthly total rainfall and monthly average humidity. The result showed that a positive correlation existed between the monthly average humidity and the infestation indexes (*C_r_*, *P_M_*, *MA* and *MI*) of *L. deliense* and *L. imphalum*, indicating that the higher the humidity, the higher the infestation indexes of *L. deliense* and *L. imphalum* (*r*: 0.727–0.807, *p* < 0.05 for *L. deliense*; *r*: 0.600–0.726, *p* < 0.05 for *L. imphalum*) (Table 3). The variation tendency of monthly average humidity was consistent with the monthly fluctuation of *L. deliense* and *L. imphalum* (Figures 5A–D). A negative correlation existed between monthly average temperature and infestation indexes (*P_M_* and *MI*) of *L. scutellare* (*r* = −0.666, *p* = 0.018 < 0.05 for *P_M_*; *r* = −0.657, *p* = 0.020 < 0.05 for *MI*), indicating that the higher the temperature, the lower the *P_M_* and *MI* of *L. scutellare* (Table 3). The variation tendency of monthly average temperature was opposite to the monthly fluctuation of *L. scutellare* (Figures 5E,F). The correlation coefficients (*r*) between the monthly total rainfall and the infestation indexes of three chigger species, however, were of no statistical significance (*p* > 0.05).

**Table 3:** Pearson correlation analysis for the relationship between infestation indexes of three vector chigger species and climatic factors (monthly average temperature, monthly total rainfall and monthly average humidity) at Waxi Village of Binchuan County, Dali Prefecture, Yunnan Province of southwest China (2020–2021).

Chigger species	Infestation indexes	Pearson correlation coefficients, *r* (*p*-values)		
		Average temperature (°C)	Total rainfall (mm)	Average humidity (%)
*L. deliense*	Constituent ratio (*Cr*)	0.378 (0.225)	0.522 (0.081)	0.727 **(0.007)
	Prevalence (*PM*)	0.393 (0.206)	0.517 (0.085)	0.807** (0.002)
	Mean abundance (*MA*)	0.372 (0.233)	0.508 (0.092)	0.727 **(0.007)
	Mean intensity (*MI*)	0.418 (0.177)	0.540 (0.070)	0.774** (0.003)
*L. scutellare*	Constituent ratio (*Cr*)	−0.490 (0.106)	−0.417 (0.178)	0.044 (0.893)
	Prevalence (*PM*)	−0.666* (0.018)	−0.485 (0.110)	−0.031 (0.924)
	Mean abundance (*MA*)	−0.494 (0.103)	−0.418 (0.176)	−0.043 (0.895)
	Mean intensity (*MI*)	−0.657* (0.020)	−0.492 (0.104)	−0.023 (0.944)
*L. imphalum*	Constituent ratio (*Cr*)	0.278 (0.382)	0.242 (0.449)	0.604 *(0.038)
	Prevalence (*PM*)	0.300 (0.343)	0.335 (0.287)	0.726**(0.007)
	Mean abundance (*MA*)	0.271 (0.394)	0.233 (0.465)	0.600* (0.039)
	Mean intensity (*MI*)	0.311 (0.325)	0.293 (0.355)	0.627* (0.029)

*The correlation coefficients are of statistical significance at 0.05 level (double tails), and **the correlation coefficients are of statistical significance at 0.01 level (double tails).

### 3.5 Niche breadths and niche overlaps of three vector chigger species

In the present study, the 12 months of a year were considered 12 temporal resource series, and each host species was regarded as a host resource series. Levins’ niche breadth (*Bi*) was used to calculate the temporal niche breadths and host niche breadths of three vector chigger species, *L. deliense*, *L. scutellare* and *L. imphalum*. The temporal niche breadths of the three chigger species were as follows: *L. deliense* (*Bi* = 0.248) > *L. imphalum* (*Bi* = 0.198) > *L. scutellare* (*Bi* = 0.168). *Leptotrombidium deliense* appeared for a longer period of time throughout the year, and it had the highest value of temporal niche breadth. The appearance of *L. scutellare* lasted for the shortest period of time in the year, and its temporal niche breadth was the lowest (Table 4). *Leptotrombidium scutellare* had a wide range of hosts (14 host species) and its host niche breadth was the highest (*Bi* = 0.268). The host ranges of *L. deliense* (11 host species) and *L. imphalum* (10 host species) were relatively low, and their host niche breadths (*Bi* = 0.099 for *L. deliense*; *Bi* = 0.079 for *L. imphalum*) were much lower than that of *L. scutellare* (Table 4).

**Table 4:** Niche breadths of three vector chigger species (*L. deliense*, *L. scutellare* and *L. imphalum*) at Waxi Village of Binchuan County, Dali Prefecture, Yunnan Province of southwest China (2020–2021).

Chigger species	No. of chiggers	Temporal niche breadths		Host niche breadths	
		Time ranges (months)	Levins’ niche breadths (*Bi*)	Host ranges (species)	Levins’ niche breadths (*Bi*)
*L. deliense*	83,008	5	0.248	11	0.099
*L. scutellare*	24,839	6	0.168	14	0.268
*L. imphalum*	24,313	5	0.198	10	0.079

**Figure 6:**
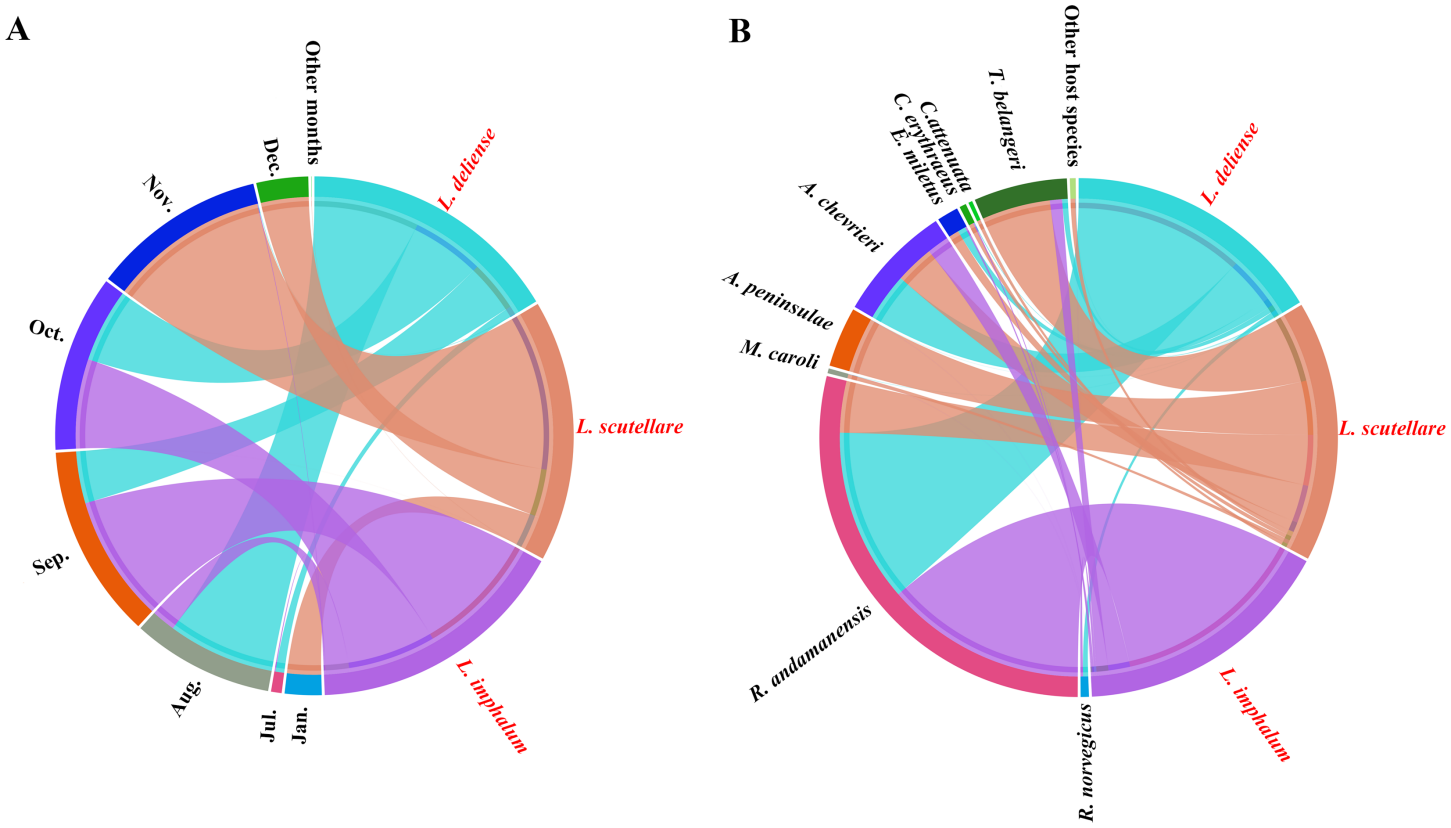
Chord diagrams for visualizing the constituent ratio (*C_r_*) distributions of three vector chigger species (*L. deliense*, *L. scutellare* and *L. imphalum*) in different months and on different host species at Waxi Village of Binchuan County, Dali Prefecture, Yunnan Province of southwest China (2020–2021). (**A**) The constituent ratio (*C_r_*) distributions of three vector chigger species (*L. deliense*, *L. scutellare* and *L. imphalum*) in different months of a year. (**B**) The constituent ratio (*C_r_*) distributions of three vector chigger species (*L. deliense*, *L. scutellare* and *L. imphalum*) on the body surface of different host species, rodents and other sympatric small mammals. The shade width in each color patch (or band) stands for the constituent ratio (*C_r_*) of corresponding chigger species.

On the basis of niche breadths, Pianka’s proportional similarity (*O_ij_*) was calculated to compare the niche overlaps between every two chigger species. It was found that *L. deliense* and *L. imphalum* had the highest values of temporal and host niche overlaps, with *O_ij_* = 0.715 for temporal niche overlap and *O_ij_* = 0.986 for host niche overlap (Table 5). Based on the constituent ratios (*C_r_*) of the three vector chigger species in different months and on different host species, a chord diagram was used to visualize the constituent ratio (*C_r_*) distributions of three vector chigger species (*L. deliense*, *L. scutellare* and *L. imphalum*) in different months and on different host species, in which the shade width in each color patch (or band) stands for the constituent ratio (*C_r_*) of corresponding chigger species (Figure 6). In the temporal distribution, *L. deliense* was mainly distributed in late summer (August) and early autumn (September and October), and *L. imphalum* mainly occurred in early autumn (September and October). *Leptotrombidium scutellare* was mainly distributed in late autumn (November) and early winter (December), which is very different from *L. deliense* and *L. imphalum* (Figure 6A). On the body surface of different host species, most *L. deliense* mites were found on the rat *R. andamanensis*, and the majority of *L. imphalum* also appeared on *R. andamanensis*. In contrast, *L. scutellare* scattered on a variety of small mammal hosts including the tree shrew *T. belangeri*, the mouse *A. peninsulae* and the rat *R. andamanensis*, which is very different from *L. deliense* and *L. imphalum* (Figure 2B).

**Table 5:** Niche overlaps between every two of three vector chigger species (*L. deliense*, *L. scutellare* and *L. imphalum*) at Waxi Village of Binchuan County, Dali Prefecture, Yunnan Province of southwest China (2020–2021).

Chigger species	Host niche overlaps (*O_ij_*)			
	*L. deliense*	*L. deliense*	*L. scutellare*	*L. imphalum*
*L. deliense*	1	1		
*L. scutellare*	0.003	0.569	1	
*L. imphalum*	0.715	0.986	0.543	1

### 3.6 Relationship between chigger infestation and scrub typhus

**Figure 7:**
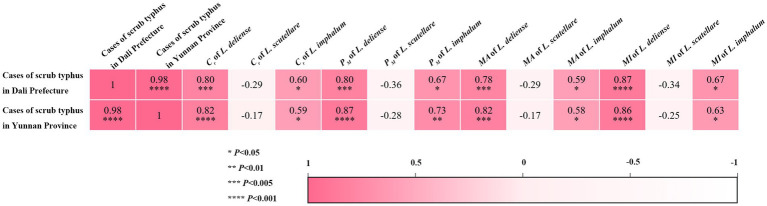
Heat map visualization for the relationships between the infestation indexes (*C_r_*, *P_M_*, *MA* and *MI*) of three vector chigger species and the cases of scrub typhus in Dali Prefecture and Yunnan Province.

Based on the published human cases of scrub typhus in Dali Prefecture and Yunnan Province from 2006 to 2022 (19), Pearson correlation coefficient was used to analyze the relationship between the monthly infestation indexes (*C_r_*, *P_M_*, *MA* and *MI*) of three vector chigger species on the hosts (rodents and other sympatric small mammals) and the monthly human cases of scrub typhus, and the correlation results were visualized by heat maps. The result showed that a positive correlation existed between the infestation indexes (*C_r_*, *P_M_*, *MA* and *MI*) of two vector chigger species (*L. deliense* and *L. imphalum*) and the scrub typhus cases (*p* < 0.05), indicating that the higher the infestation indexes of the two vector chiggers, the higher the scrub typhus cases. A slight negative correlation existed between the monthly infestation indexes (*C_r_*, *P_M_*, *MA* and *MI*) of *L. scutellare* and the scrub typhus cases, but the correlation coefficients (*r*: from −0.17 to −0.36) were of no statistical significance (*p* > 0.05) (Figure 7).

## 4 Discussion

Of the three vector chigger species studied in the present study, *L. deliense* is the most important vector of scrub typhus over the world and it is the main vector of the disease in tropical and subtropical regions (4, 30, 32). *Leptotrombidium scutellare* is also very important and it is a main vector of the disease in some temperate regions (33, 34). Although *L. imphalum* is not as important as *L. deliense* and *L. scutellare* in the transmission of scrub typhus, it is also an effective vector of the disease in some local regions (20, 23, 35, 36). In addition, *L. scutellare* is also a potential vector of HFRS (5, 6). The seasonal fluctuation of vector chiggers usually has a direct impact on the seasonal incidence of chigger-borne diseases like scrub typhus (18, 37, 38), and therefore it is of medical significance to study the seasonal dynamics of vector chiggers in the foci of scrub typhus. In the present study, the survey site (Waxi Village of Binchuan County) is a focus of scrub typhus and HFRS (11, 19, 20), and it is located within the territory of Dali Prefecture in Yunnan Province where both scrub typhus and HFRS are prevalent with high incidence of the diseases (19, 20). Although there were no monthly cases of scrub typhus available at the survey site, the monthly cases in Dali Prefecture and Yunnan Province were obtained instead. The result showed that a positive correlation existed between the monthly infestation indexes (*C_r_*, *P_M_*, *MA* and *MI*) of two vector chigger species (*L. deliense* and *L. imphalum*) and the monthly human cases of scrub typhus in Dali Prefecture and Yunnan Province where the survey site is located (Figure 7). The positive correlation between the monthly infestations of vector chiggers and the monthly scrub typhus cases suggests that the seasonal fluctuation of vector chiggers may influence the seasonal incidence of scrub typhus (18, 37, 38).

As three dominant chigger species at the survey site, *L. deliense*, *L. scutellare* and *L. imphalum* can ectoparasitize a wide range of small mammal hosts, and *L. scutellare* had much wider host niche breadth than *L. deliense* and *L. imphalum*, indicating the low host specificity of vector chiggers (Table 4). The low host specificity of vector chiggers is conducive to their transmitting the pathogens of scrub typhus and HFRS among wild animals (especially rodents), and even from wild animals to humans (39–41). The coexistence of these three vector chigger species as the dominant mite species at the same survey site further increases the potential risk of the zoonosis transmission and focus persistence.

The distribution of chiggers and other ectoparasites is often influenced by a series of environmental factors, including different latitudes, longitudes, altitudes, and geographical landscapes (14, 27, 39). In order to eliminate the interference of these environmental factors, it is usually necessary to select a fixed survey site and conduct field investigations at different time period when studying the seasonal dynamics of chiggers and other ectoparasites (37, 42, 43). Therefore, the present study selected Waxi Village of Binchuan County, Dali Prefecture, Yunnan Province of southwest China as the fixed survey site for studying the seasonal dynamics of vector chiggers. Different species of chigger mites may have different types of seasonal fluctuations, and sometimes the same chigger species may also show different types of seasonal fluctuations in different geographical regions (1, 18, 44). The various seasonal fluctuation patterns of chigger mites can be summarized into several types such as summer type, summer–autumn type, autumn type, autumn–winter type, and winter type in China (1, 41, 42, 45). The results of the present study indicate that *L. deliense* and *L. imphalum* have similar seasonal fluctuation patterns, belonging to the “summer-autumn type.” The seasonal fluctuation pattern of *L. scutellare*, however, is obviously different from those of *L. deliense* and *L. imphalum*, and it belongs to the “autumn-winter type” (1, 41, 42, 45). Of the three vector chigger species, *L. deliense* and *L. imphalum* had the highest values of temporal and host niche overlaps (Table 5, Figure 6). The highest overlaps of temporal and host niches between *L. deliense* and *L. imphalum* indicates that these two chigger species have similar seasonal fluctuation patterns and similar tendency in host selection. *Leptotrombidium scutellare*, however, has different seasonal fluctuation pattern and host selection from *L. deliense* and *L. imphalum*.

A previous investigation at Jingha Village of southern Yunnan showed that *L. deliense* had two peaks in July of summer and October of autumn for its seasonal fluctuation with the highest peak in July. Although the seasonal fluctuation pattern of *L. deliense* at Jingha of southern Yunnan also belongs to “summer-autumn type,” its highest peak in July was 1 month earlier than that of the mite (August) at Waxi of Binchuan County in the present study (Table 2, Figure 3) (16). *Leptotrombidium deliense* tends to live in environments with high temperature and humidity (16, 32). In Yunnan Province of southwest China, the annual average temperature and relative humidity in the southern areas with low latitudes are usually higher than those in the northern regions with high latitudes, and the hottest season with high relative humidity in the south also comes earlier than that in the north. Waxi (25^°^43′ N) of Binchuan County in the present study is located at the northwest of Yunnan (Figure 1), and it is far away from Jingha (21^°^50′ N) at the southernmost tip of Yunnan, and this may explain why the highest peak of *L. deliense* at Jingha comes one month earlier than that of the mite at Waxi in the present study (16). Some investigations from other provinces of China and other countries (or regions) have shown that *L. deliense* may show different seasonal fluctuations in different countries and regions. In Guangzhou of southern China, *L. deliense* occurred throughout the year and maintained a high abundance level from May to November. In Fujian of southeastern China, *L. deliense* began to appear in small numbers in April, reached a peak from June to August, and then decreased after September, and it was almost invisible in winter (1). In Tamil Nadu of India, there was a high mean abundance of *L. deliense* from October to December, peaking in November (18). The infestation index of *L. deliense* was high from July to November and peaked in October in Gorakhpur district, Uttar Pradesh of India (46). Although *L. deliense* showed different seasonal fluctuations in different countries and regions, its peaks in summer or autumn are similar to the result of the present study. The results suggest that *L. deliense* tends to occur in large numbers during the hot and humid summer and autumn (1, 18, 46). To date, there have been no other reports on the seasonal fluctuation of *L. imphalum*, and the present study reported the seasonal dynamics of *L. imphalum* for the first time.

The variation tendency of monthly average humidity was consistent with the monthly fluctuation of *L. deliense* and *L. imphalum*, and a positive correlation existed between the humidity and infestation indexes of these two vector chigger species (*p* < 0.05). The results may reflect the impact of the monthly average humidity on the seasonal fluctuation of *L. deliense* and *L. imphalum*, and the humidity may be one of the important climatic factors affecting the seasonal dynamics of these two chigger species (1, 35). The negative correlation between the monthly average temperature and *L. scutellare*’s infestation indexes (*p* < 0.05) may reflect the impact of temperature on the seasonal fluctuation of *L. scutellare* (Table 3, Figure 5), indicating that *L. scutellare* prefers to select cold seasons with low temperature. In Fujian of southeastern China, *L. scutellare* began to appear in small numbers in October of autumn, reached a high abundance level from December of the previous year to February of the following year in winter, and decreased in April of spring, and it was not found after May (1). *Leptotrombidium scutellare* was a dominant species of chigger mites in Japan, and it occurred from October of the previous year to April of the following year and peaked in January of winter (47). The seasonal fluctuation pattern of *L. scutellare* in the present study (autumn-winter type) is consistent with the previous reports, and it suggests that *L. scutellare* tends to occur in large numbers during the cold and dry late autumn and winter (37, 42).

The Chinese Center for Disease Control and Prevention (CDC) had released a document titled “Technical Guidelines for the Prevention and Control of Scrub Typhus.” This document provided a series of suggestions and technical guidelines for the standardized diagnosis and treatment of scrub typhus, vector surveillance and control, as well as population protection (48, 49). In the actual campaign of surveillance and control, however, scrub typhus and its vector chiggers have long been neglected. Nowadays, as the epidemic areas of scrub typhus in China continue to expand, local authorities have realized the potential threat of scrub typhus and its vector chiggers to public health (11, 19, 50). The results of the present study will provide important scientific data and guidance for the future surveillance and control of scrub typhus and vector chiggers.

As mentioned above, a series of environmental factors can influence the distribution of chiggers and other ectoparasites (14, 27, 39). In order to eliminate the interference of non-seasonal factors and to objectively demonstrate the seasonal dynamics of chiggers and other ectoparasites, it is essential to fix a survey site to do a year-long field investigation (37, 42, 43). The result from a fixed survey site, however, can only represent the situation at that location and cannot reflect the varying conditions in other geographical locations beyond the survey site. To understand the seasonal dynamics of chiggers and other ectoparasites in different geographical areas, different fixed survey sites must be chosen for a year-long field investigation (e.g., a consecutive 12-month survey). Due to the lack of sufficient human resources and financial support, the present study only conducted a consecutive 12-month survey at one fixed survey site (Waxi), which cannot reflect the varying conditions in different geographical locations of Yunnan Province or southwest China. To explore the varying seasonal dynamics of chiggers in different geographical areas, a few fixed survey sites are recommended in future studies.

## 5 Conclusion

The three vector chigger species, *L. deliense*, *L. scutellare* and *L. imphalum*, can ectoparasitize a wide range of small mammal hosts with low host specificity, and rodents (Rodentia) are their main hosts. The coexistence of the three vector chigger species as the dominant mite species at the same survey site, together with their low host specificity, increases the potential risk of transmission and focus persistence of relevant mite-borne zoonoses. *Leptotrombidium deliense* and *L. imphalum* have similar seasonal distribution patterns (summer-autumn type) and host selection, and *L. scutellare* has a different type of seasonal fluctuation, autumn-winter type. The temporal and host niches of *L. scutellare* are very different from those of *L. deliense* and *L. imphalum*. The temperature and humidity may be important climate factors that affecting the seasonal dynamics of vector chiggers. The seasonal fluctuations of vector chigger populations are associated with the seasonal incidence of scrub typhus.

## Data Availability

The original contributions presented in the study are included in the article/supplementary material, further inquiries can be directed to the corresponding author.

## Footnotes

^1^ www.xihe-energy.com
